# Cochlear implant in postlingual children: functional results 10 years after the surgery

**DOI:** 10.1590/S1808-86942012000200016

**Published:** 2015-10-20

**Authors:** Liege Franzini Tanamati, Maria Cecilia Bevilacqua, Orozimbo Alves Costa

**Affiliations:** a MSc. Speech and Hearing Therapist at the Núcleo Ouvido Biônico of the Hospital Samaritano; b Full Professor – University of São Paulo. Coordinator of the HRAC Audiological Research Center – USP – Bauru and the Núcleo do Ouvido Biônico – Hospital Samaritano – SP); c Full Professor – University of São Paulo. Coordinator of the Núcleo do Ouvido Biônico – Hospital Samaritano – SP

**Keywords:** adolescent, child, cochlear implants, follow-up studies, young adult

## Abstract

The benefits of cochlear implants (CI) for communication skills are obtained over the years. There are but a few studies regarding the long-term outcomes in postlingual deaf children who grew up using the electronic device.

**Aim:**

To assess the functional results in a group of postlingual children, 10 years after using a CI.

**Methods:**

Ten postlingual deaf children, implanted before 18 years of age, participated in this study. We assessed: sentence recognition and speech intelligibility. We documented: device use and function and the patient's academic/occupational status. Study design: series.

**Results:**

The mean scores were 73% for sentence recognition in silence and 40% in noise. The average write-down intelligibility score was 92% and the average rating-scale intelligibility score was 4.15. There were no cases of device failure. Regarding educational/vocational status, three subjects graduated from the University. Five quit education after completing high school. Eight subjects had a professional activity.

**Conclusion:**

This study showed that cochlear implantation is a safe and reliable procedure. The postlingual profoundly hearing-impaired children after 10 years of CI use developed satisfactory levels regarding speech perception and intelligibility, and completed at least high school and were inserted in the labor market. Clinical Trials Registry: NCT01400178.

## INTRODUCTION

Cochlear Implants (CI) have been broadly accepted as the most efficient technological resource among the many alternatives to treat patients with severe to profound bilateral sensorineural hearing loss – who do not have satisfactory results using individual sound amplification devices. Since 1990, when the FDA approved CI for children, a growing number of them have been implanted world wide^1^, ^2^, ^3^.

There is still no consensus as to results stability concerning the hearing skills developed by children with CI, nor about the time needed to develop such skills, which may take years, thus the importance of long term follow up studies^4^, ^5^.

It is expected that CI users reach speech understanding, even in adverse communication situations, and develop it in an intelligible way, have a satisfactory perception of music and an effective communication on the telephone^6^. These goals can be accomplished by some users; nonetheless, there still is a great variability in individual results – as per reported by implant centers throughout the world. Numerous studies have been carried out in order to find the factors which influence speech and language development after the implant^7^.

One of the important factors which interfere in the long term benefits the child will have after the CI is associated to the age at which the child develops the hearing impairment (HI). It is known that children with normal, or near-normal hearing before the HI sets in, tend to have better performance when compared to those who are born deaf. Hearing input associated with the neural plasticity and the linguistic skills developed prior to the HI can be useful in helping children interpret the auditory information provided by the CI^2^, ^8^.

There are very few studies in the literature describing the long term results in postlingual HI children, those who grew up using a CI. The studies describe the results obtained only in the first months of using the device, assessing the communicative skills alone. These few studies show that although the CI is an unchallenged successful method to treat postlingual HI children, its results vary considerably and the long term benefits cannot be predicted for the users^3^.

In the studies which compared the performances of pre, peri and postlingual children, results showed that the children with perilingual or postlingual HI reached the best performances and more accelerated development of their auditory skills and speech production than the prelingual HI children, and in many cases one can notice patterns which are similar to those presented by postlingual adults implanted with a CI^1^, ^2^, ^8^, ^9^, ^10^.

The goal of the present study was to document the performance achieved by children with postlingual HI, as far as speech perception, speech intelligibility and academic/occupational level are concerned, as well as the complications arising from the device after 10 years of cochlear implant use.

## MATERIALS AND METHODS

The present study was approved by the Ethics Committees of two centers, which research protocol numbers were 0685/08 and 186/2008, respectively.

This study was cross-sectional, involving the first group of children with profound bilateral sensorineural HI, consecutively implanted between 1990 and 2000, in a reference pediatric center in our country – regarding CI surgery and follow up.

Only postlingual HI children submitted to the CI before 18 years of age were selected to the study. The duration of device use was of, at least, ten years. Four individuals were taken off the study because they did not come to the hospital at the appointed date. The demographic data of the 10 participants in the study are listed on Table 1. Six participants were users of the *Nucleus 22* (Cochlear) device, the *Freedom* speech processor and SPEAK strategy. Three participants used the C40+ (Med–El) device, Tempo + speech processor, and CIS strategy. One participant used the Bipolar/standard 1.2 (*Advanced Bionics*) implant, *S-Series processor, with* MPS strategy. The mean age of HI onset was 8.9 years (SD=+ 3.2; between 5.8 and 14.8 years). In the CI group, the mean age was 10.9 years (SD= +3.8; between 5.4 and 16.1 years) and, upon assessment, the mean age corresponded to 24.3 (SD=+ 4.4; between 16.5 and 31.3 years).

**Table 1:** Demographic data of the postlingual children.

Age (years)					
Participant/Device	Upon HI onset	Upon CI	Upon assessment	Duration of use	Etiology
1/N22	6.3	7.0	23.4	16.3	Meningitis
2/N22	14.8	15.3	31.3	15.11	Meningitis
3/N22	6.0	8.1	23.2	14.0	Mumps
4/N22	10.0	13.5	27.5	13.11	Idiopathic
5/N22	9.0	11.11	25.3	13.3	Meningitis
6/N22	7.0	9.10	22.5	12.6	Meningitis
7/C40+	14.0	14.9	25.11	11.1	Ototoxic
8/C40+	7.1	8.7	19.3	10.7	Encephalitis
9/C40+	5.8	5.4	16.5	11.0	Meningitis

10/Bipolar/standard 1.2	9.0	16.10	29.2	12.3	Mumps

The participants were submitted to the procedures included in this study, which were: auditory performance assessment test in relation to speech perception; speech intelligibility assessment; questions in order to collect information about the use and functioning of the device and the person's academic/occupational situation.

### Speech perception

The participant's speech perception was assessed by the recognition of dissyllable words, created by Lacerda^11^. The list with 25 phonetically balanced dissyllable words (cvcv structure) was employed with the patient in a 2mx2m sound-treated booth, presented at a fixed level of 60 dB SPL. The participant was seating 1 meter away from the speaker, at 0^º^ Azimuth.

The phrases recognition was assessed by means of the Hint test (*“Hearing in Noise Test”*). The version of the Hint test employed in this study was the “Hint for Windows, version 7.2” (Hint Manual^12^). The equipment utilized for the Hint test application was: CD of the Hint Pro software and calibration, USB cables, speaker and a desktop computer. This equipment was set up in a sound-treated room.

Since all the participants of the current study used the CI device, the Hint test was used only in the free field, with frontal noise presentation. The procedure lasted for approximately 15 to 20 minutes. The participant was positioned in front of the speaker, one meter away from it, instructed that it was a procedure to test his/her ability to perceive speech in silent or noisy environment. During the test, the participant was asked to remain in the same position in his/her chair, without moving neither the head nor the body, and should remain in front of the speaker. The patient was told there would be a man reading a phrase and he/she should repeat everything that was said, even if his voice seemed very soft and, even if only part of a sentence had been understood.

We employed two lists of phrases randomly selected by the software: one list in silence and another in the presence of frontal noise. Under silence, the phrases were presented at a fixed level of 60 dB and; in the case of frontal noise, it was presented in relation to a fixed signal to noise ratio of +10 dB. For each situation, we then obtained the total number of words repeated correctly. The final result was expressed in percentage.

In order to complete the hearing perception assessment of the users, during the interview with the participant and a family member, we investigated telephone use. We asked the participant whether or not he/she was able to understand a communication through the telephone, preference for a specific type of speaker, and whether the preference was for the land line or the cell phone.

### Speech intelligibility

The speech intelligibility assessment of each participant was based on three stages: material building, application of the procedure (recording the phrases read by each participant) and recording assessment by the examiners. These stages were based on the studies carried out by Monsen^13^ and Peng et al.^14^.

Because of not having a material in Portuguese to be specifically used in the assessment of speech intelligibility, for the present study it was necessary to build a written material, made up by phrases, which would be used by the participants for reading and recording. The material was prepared based on the phrases developed by Murari^15^, in order to assess the auditory perception of the hearing impaired children. Of the 494 phrases proposed by this author, we selected only 18 phrases, making up a total of 100 key-words (Attachment 1).

Attachment 1 **Material built to assess speech intelligibility: list of phrases for the training and the test (Murari phrases, 2004).**

*Training phrases list:*

1. She went to complain for my mother.
2. I had a broken leg.

*Test phrases list:*

1. My mother went to the store to buy milk.
2. I don't know what the name of the fruit is.
3. The other day he fought with my father.
4. My mother did not allow going to the street.
5. My brother always wants to go there.
6. I went there looking for my brother.
7. The woman went back to her home.
8. My father went to work at night.
9. Daddy went to buy guarana and beer.
10. She took the girl there to the sofa.
11. I parked there, at the beach again.
12. I can speak a little English.
13. First we went to see the monkeys.
14. I went home and took the tennis shoes off.
15. Tomorrow morning I am going to the beach.
16. He had broken my bed.
17. I do not have time to play.
18. On the other day I left home.

Each participant was asked to read the 18 phrases, one by one, which were recorded. The recording of the phrases was carried out by the “*Sony Sound Forge Software*” 9.0e, 2007 (Sony Creative Software Inc.). The software was installed in an HP Pavilion dv4–1230br laptop computer. For recording purposes, we used the “*MicroBoom*” type of external microphone, from Phonak. In order to improve and standardize the audio quality in the analysis, we used the functions available in the *Sound Forge* version 9.0 software. The file from each participant was saved in an individual directory, and randomly selected to be presented to the examiners, who carried out the assessment of the speech intelligibility of the participants in this study.

The pre-requisites adopted to be judged were the following: normal hearing; completed high school education and no experience with the speech of hearing impaired people. Each examiner completed his/her listening task in a silent room. The phrases were presented using a laptop computer connected to a TDH source. The examiner controlled the presentation of the phrases, and there were no time restrictions for the transition of each phrase.

Each examiner assessed the speech intelligibility of only one participant. Nonetheless, the same list of phrases recorded for each participant was assessed by two examiners.

The participants' speech intelligibility was assessed by two methods: the writing transcription method, that is, the examiner had to write down what he/she understood from the phrase presented, and by the intelligibility classification method in levels belonging to a scale – the Intelligibility Scale.

The examiners were instructed to hear each phrase twice. Each examiner had one blank sheet, only with the number of each phrase (1 to 18). The examiner was asked to transcribe the phrase in written, in each one of the presentations. The word was considered correct when there was a phonemic correspondence between the written transcription and the expected key-word. Each correct key-word was scored as a correct answer. For each transcribed phrase, both in the first as in the second presentation, the examiner was also asked to classify the participant's speech in an ordinal scale of intelligibility, with levels between 1 and 5; level 1 corresponds to the “unintelligible speech” and, level 5 corresponds to “a totally intelligible phrase”, the examiner should mark the level which best corresponded to his/her opinion about the intelligibility of the speech presented.

The final result was calculated based on the analysis of inter-examiner responses, in other words, we analyzed the results between the two examiners. The mean percentages of the transcription and the scale median value were compared between the two participants, for the four presentations.

### Device functioning/academic and occupational situation

The participant and a family member were interviewed using a support material, which was made up of an interview script. The aim of such script was to guide the collection of information, stressing the specific topics associated with the use and functioning of the device and the academic and occupational situation of each participant.

## RESULTS

### Speech perception

The speech perception results, after over 10 years of CI use are described on Table 2. All the participants were able to recognize speech without the help of orofacial reading. The mean value of the hearing recognition performance for dissyllable words was equal to 61%, varying between 24% and 84%. As to the Hint phrases recognition in silence, the mean value of correct answers was equal to 73%, and 40% under noise. Speech recognition in noise reduced, in average was 33.3% when compared to the silence situation.

**Table 2:** Detailed results obtained from the 10 postlingual participants.

*% phrases Hint*				*Intelligibility*			
P	Dissyllables (%)	Silence	Noise	Telephone	Transcription (mean)	Scale	Academic/occupational level I
1	42	68.75	12.37	Any person/Cell phone	62.25	2	Finished high school. Works.
2	24	41.30	3.45	Familiar/Home phone	99.5	5	Finished college. Works and is in a specialization program.
3	84	92.78	39.77	Any person/Cell phone	93	4	In college. Works.
4	76	85.71	58.33	Any person/Cell phone	100	5	Finished college. Works.
5	84	84.27	53.60	Any person/Cell phone	100	4.5	Finished high school. Works
6	76	94.38	53.33	Any person/Cell phone	97.25	5	Finished high school. Works.
7	56	51.68	29.67	Any person/Home phone	99.5	5	Finished college. Works.
8	48	89.13	68.13	Any person/Cell phone	100	5	Finished high school. Does not Work.
9	80	83.15	66.33	Any person/Home phone or Cell phone	99	4	In high school. Does not Work.
10	40	39.08	12.24	Any person/Home phone or Cell phone	69.5	2	Finished high school. Works.

As to telephone use, all the participants reported they used the telephone and understood the speaker. Only one participant reported having difficulties understanding strangers, preferring to speak to family members. The others reported they could understand anyone on the telephone. Six participants preferred the cell phone, two reported they could understand better a landline phone and two participants used both the landline as well as the cell phone.

### Speech intelligibility

After 10 years of CI use, the results obtained by the transcription method showed that, in average, the percentage of words correctly transcribed was 92% (SD=+14; varying between 62.2% and 100%). Table 2 showed the results of the intelligibility assessment for each participant.

As to the results obtained by means of the intelligibility scale, the speeches from eight participants were classified between levels 4 and 5 of the scale, in other words, levels corresponding to the totally intelligible speech. The speeches of only 2 participants were classified in the level 2 of the scale. These participants had results between 60% and 70%, according to the transcription method. The participants (n=8) classified between categories 4 and 5 had performances between 90% and 100% in the transcription method.

### Device use and functioning

Considering the background of the 10 participants described in this study, we recorded only one case of complication with the internal device. Participant P3's intracochlear electrodes bundle extruded after 4 years of device use. It was necessary to do a second surgical procedure in order to reposition the device.

In the remaining nine cases, we did not find other complications with the internal device. There were no episodes of internal device failure along the more than 10 years of CI use.

As far as use is concerned, the ten participants reported always using the same device. All reported using it every day, over 10 hours a day. When asked about situations in which, sporadically, they did not use the device, two participants reported that, in the weekends, they did not place the device immediately after waking up. Two other participants mentioned that the most common situation, when they did not use the external device, was during the night, after the shower.

### Academic/occupational situation

At the time of the interview, there was only one participant with less than 18 years of age, who was in his sophomore year of a regular high school. One participant, with 23 years of age, was in college. Five participants finished high school: three were studying to take the University Entrance Exam and two had stopped to study. Three participants finished college. Among them, one participant was taking a specialization program. All the participants were going to or had gone to only regular schools throughout their academic trajectories.

Eight of the ten participants were working at the time of the interview. Of these participants who were employed, seven got a job through a quotas system, in other words, in spots saved for hearing impaired individuals. One participant was working in a family business. Their wage varied between R$ 400.00 and R$ 2,000.00.

## DISCUSSION

Studies carried out with children, after numerous years using the CI, enable a better understanding concerning the extension of the benefits reached by the users concerning the development of their hearing and language skills^16^, ^17^. To study the performance of the children implanted for a long time is also vital, since it enables a better explanation about the long term effects of the electrical stimulation of the auditory system, enabling healthcare professionals to provide more detailed information about the long term stability of the results to future CI candidates and their families^5^, ^18^, ^19^.

The present study reported the results reached by 10 children with postlingual HI, after over 10 years using the CI, in relation to speech, speech intelligibility, the use and working of the device, vis-á-vis speech perception, speech intelligibility, and the use and functioning of the device and the academic and/or occupational level.

There is a scarce number of studies in the literature regarding the results obtained from postlingual HI children submitted to CI, making it difficult to compare the studies. In the first years using the CI, studies comparing the performance from pre and postlingual children showed considerable better results among those children whom HI onset happened after four years of age^1^, ^2^, ^10^. Such findings emphasized that the proper auditory sensory input and the previous linguistic skills, developed before HI onset, were determining for the children to interpret and better use the information provided by the CI.

In relation to the auditory perception, after over 10 years using the CI, all the participants in this study were able to perform the tests in an open set. All the cases of implanted postlingual children assessed in the literature found similar results^1^, ^9^, ^10^; nonetheless, the duration of use of the children assessed varied considerably among the studies, as well as the methodology employed to assess the auditory perception. In the study carried out by Mitchell et al.^2^, only one of the 14 postlingual children assessed did not reach the level of auditory skill necessary to perform the auditory recognition tests in an open set. This child, whose HI was caused by meningitis, was submitted to CI after the 4 years of age. The authors did not report on the other characteristics of the child.

In the present study, the mean percentage of correct answers in the dissyllable word recognition test was equal to 61%. Better results (>93%) were found in the study carried out by Mitchell et al.^2^. The authors also employed a list of words to assess the auditory recognition of the postlingual children; nonetheless, they used monosyllable word lists (PBK – “*Phonetically Balanced Kindergarten words test*”).

After over 10 years using the device, the best results were found for the recognition of phrases in silent environments. The mean percentage of correct answers for Hint phrases in silence was equal to 73%. Similar results were found by Kiefer et al.^1^ and Mukari et al.^9^. Because of the prior auditory experience, before the HI onset, in many cases the postlingual children can reach similar patterns to those found by postlingual adult users of CI^8^, ^10^, ^20^. In the study carried out by Firszt et al.^21^ with postlingual adults, the mean number of correct answers for the Hint phrases in silence corresponded to 85%.

Considering the different situations and day-to-day environments, to assess the auditory perception in the presence of noise is fundamental in order to explore the real difficulties CI users experience in their daily lives. The results obtained in this study showed that, in fact, a greater difficulty in recognizing phrases in the noise was observed in all the patients. In average, there was a 33% reduction in the performance of the participants, when compared to the situation upon the presentation of phrases in silence and in noise. The results found in this study may be associated to the fact that all the participants received an indication of CI at a time when the indication criteria were, in a given way, more conservative, from the audiological standpoint. Notwithstanding, this group of children received older generation internal devices, with less sophisticated speech coding strategies.

Despite the benefits provided by the CI device for the capturing sound by the device users, to perceive speech in noisy environments continues to be one of the most challenging situations, even for implanted adults. Studies have shown that the adverse effects of noisy environments may be reduced by means of the use of advanced technological systems associated with the CI. New pre-processing strategies, Frequency Modulation (FM) systems, directional microphones, larger Input Dynamic Ranges (IDR) are example of technological resources currently available to attempt to minimize the effects of noise for the speech performance of implanted children^22^, ^23^. Considering such technological innovation and resources, it is expected that children implanted with more current devices may have a better auditory performance in day-to-day situations.

As far as telephone use is concerned, all the participants reported understanding speech through the telephone. It is a significant result concerning greater independence and practicality during daily routine activities. Only one participant, because he reported difficulties in understanding different speakers, restricted the use of the telephone to known persons (family members and close friends) – a fact which was also observed by other studies with prelingual children after more than 10 years of using the device^24^, ^25^.

After more than 10 years using the device, most of the participants (n=8) scored higher than 90% in the transcription method, and the mean percentage of correct answers was equal to 92%. In the study carried out by Hiraumi et al.^26^, the results from the speech intelligibility assessment in ten postlingual children, following the phrase transcription method, was equal to 80.4%. Differently from the present study, the participants of the Hiraumi et al.^26^ study had been using the CI device for less time. At the time of assessment, the duration of CI use was, at least, 6 months. The authors did not report the maximum duration of device use.

In relation to the results from the intelligibility scale, the speech from eight participants was classified between the levels 4 and 5 of the scale, which represented levels corresponding to a totally intelligible speech. The two participants who had a percentage below 70% in the transcription method were the same who had the worst results in the scale classification, being classified in the scale 2 of intelligibility. Similar results were found by Mitchell et al.^2^. Among the 14 children assessed, 11 reached the level of speech considered intelligible to all the listeners or to listeners with less experience with the speech of hearing impaired individuals. Two children had intelligible speech only to one listener with experience with the speech of hearing impaired individuals and one child had an intelligible speech.

Although the participants in the present study already had intelligible oral language before HI onset, the results obtained, through the transcription method and intelligibility scale, showed that the speech of the participants remained intelligible to listeners who had no experience with the oral communication of HI patients. The hearing *feedback*, enabled through CI stimulation, proved it was effective to keep intelligible the participants' speech, even after years using the device.

Of the 10 participants in this study, there was only one case of complications with the internal device. There were no episodes of internal device failures along more than 10 years of CI use. Failure cases were reported by some studies in the literature, assessing prelingual children in the long run. Nine failure cases (30%) were reported by Beadle et al.^24^, during the CI use period, between 10 and 14 years. Eleven cases (13.4%) were described by Uziel et al.^25^ with children implanted after 10 years using the device. The authors stress that the cases of complications involving the internal device, peaking or not with the need to re-implant the device, must be considered by CI programs, which must prioritize the periodic follow up of CI users, with the aim of monitoring the status and the functioning of the internal device and, then, promoting actions to minimize the inevitable tension inherent to situations of complications with the device.

The results regarding device use were excellent and are in agreement with other studies in the long term^24^, ^25^. All the participants in the study reported using the device effectively. The participants used the speech processor every day for more than 10 hours. The fact that the teenager and the young adult chose to use the device indicates the value the user assigns to the device, as well as the benefits arising from its use.

According to Summerfield & Marshall^27^, the course of time for the development of the entire cascade of benefits reached by the CI users encompasses, at least, 20 years. Specifically, the long term benefits expected for CI users include: independence in social relations, academic improvements in higher levels, professional opportunities, an even greater improvement in quality of life. The information obtained from this study showed that, after more than 10 years using the CI, all the participants were properly adjusted to their daily life routines, finishing their studies, or had a job. Beadle et al.^24^ and Spencer et al.^18^ also found encouraging results in relation to the occupational and academic level of children who grew up using the CI.

## CONCLUSION

This study showed that the CI device, in fact, is a safe and reliable procedure, even years after the surgery. There were no cases of device failure. The children with postlingual hearing disorder reached, after 10 years, functional results in relation to auditory perception and speech intelligibility. The participants of the present study finished, at least their higher education and were employed.
